# Effect of Chinese Herbal Medicine on Pregnancy Outcomes in IVF Patients with Low Ovarian Reserve: A Predictive Model

**DOI:** 10.3390/medicina61091571

**Published:** 2025-08-31

**Authors:** Meng-Hsing Wu, Shu-Chiu Wang, Pei-Fang Su, Liang-Miin Tsai, Po-Ming Chen, Chia-Jung Li, Hsing-Ju Wu, Chiung-Hung Chang

**Affiliations:** 1 Department of Obstetrics and Gynecology, College of Medicine, National Cheng Kung University, Tainan 701, Taiwan; mhwu68@mail.ncku.edu.tw; 2 Department of Traditional Chinese Medicine, Tainan Municipal Hospital, Tainan 701, Taiwan; wangsc8899@gmail.com; 3 Department of Statistics, National Cheng Kung University, Tainan 701, Taiwan; pfsu@ncku.edu.tw; 4 Department of Internal Medicine, Tainan Municipal Hospital, Tainan 701, Taiwan; tsailm0126@gmail.com; 5 Research Assistant Center, Show Chwan Memorial Hospital, Changhua 500, Taiwan; yaoming9@yahoo.com.tw (P.-M.C.); hildawu09@gmail.com (H.-J.W.); 6 Department of Nursing, Central Taiwan University of Science and Technology, Taichung 406, Taiwan; 7 Department of Obstetrics and Gynaecology, Kaohsiung Veterans General Hospital, Kaohsiung 813, Taiwan; nigel6761@gmail.com; 8 School of Chinese Medicine for Post-Baccalaureate, I-Shou University, Kaohsiung 840, Taiwan

**Keywords:** low ovarian reserve, in vitro fertilization, Chinese herbal medicine, prediction model, live birth rate

## Abstract

*Background and Objectives*: Live birth rates in women with diminished ovarian reserve (AMH < 2.0 ng/mL) undergoing in vitro fertilization (IVF) remain under 20%. While Chinese herbal medicine (CHM) is often used to treat infertility and support IVF, its effectiveness in this group is unclear. This study assessed live birth outcomes by treatment type and patient characteristics. *Materials and Methods*: We retrospectively analyzed 240 infertile women with low ovarian reserve and partners with normal sperm. Of these, 63 were in the control group, 118 received CHM only, and 59 received IVF with CHM. Logistic regression and a nomogram were used to assess live birth outcomes. *Results*: The live birth rate was 5% in the control group, 32% in the CHM-only group, and 42% in the mixed treatment group. Univariate analysis showed that both treatment type and patient age were significantly associated with live birth outcomes. In multivariate logistic regression, CHM-only treatment was significantly associated with an increased likelihood of live birth (odds ratio [OR]: 8.98, *p* = 0.001), as was the mixed treatment group (OR = 18.77, *p* < 0.001), with the control group as the reference. Women aged ≤37 had significantly higher live birth rates (OR = 3.24, *p* = 0.018), using those over 37 as the reference group. A predictive model was developed based on age, AMH level, and treatment type, achieving an area under the curve (AUC) of 0.756 for predicting live birth probability. *Conclusions*: This clinical prediction model offers guidance for couples and clinicians on pregnancy outcomes in low ovarian reserve patients treated with CHM alone or alongside IVF.

## 1. Introduction

The term “ovarian reserve” refers to the number of oocytes remaining in a woman’s ovaries [1,2]. A woman’s fertility largely depends on her ovarian reserve, which plays a critical role in achieving pregnancy [1]. Ovarian reserve tests (ORT) commonly used to assess this include measurements of follicle-stimulating hormone (FSH), inhibin B, antral follicle count (AFC), and anti-Müllerian hormone (AMH) [3,4]. AMH is a glycoprotein hormone secreted by granulosa cells of the ovaries and continues to be produced until menopause [5]. The normal reference range of serum AMH is approximately 2.0 to 6.8 ng/mL, regardless of the menstrual cycle phase [6]. AMH levels decline with age and are clinically used as a stable marker of ovarian reserve and a reliable predictor of oocyte quantity [7]. Therefore, this study used AMH to evaluate ovarian function. Low ovarian reserve (LOR) was defined as a serum AMH level below 2.0 ng/mL [8,9]. Patients with LOR share clinical characteristics with those diagnosed with diminished ovarian reserve (DOR), which is typically defined as a serum AMH level ≤0.5–1.1 ng/mL [10].

LOR, as well as DOR, is characterized by a decrease in quantity and quality of ovarian follicles in reproductive-age women [4,11] and is associated with reduced reproductive capacity, which can cause infertility and premature menopause [10,12]. As a result, women with LOR often seek assisted reproductive technology (ART) to improve their chances of getting pregnant. According to the 2017 American assisted reproductive technology national summary report, DOR (31.6%) was the most commonly reported reason for ART [13]. In vitro fertilization (IVF), one of the ART procedures, is the most commonly used for couples with infertility [14,15].

Nomograms are effective tools in reproductive medicine, providing personalized predictions of pregnancy outcomes based on clinical factors. Ballester et al. (2012) developed a model for ICSI-IVF in endometriosis patients, while Zhang et al. (2022) applied nomograms to frozen embryo transfers, highlighting the role of endometrial and hormonal factors [16,17]. Zhu et al. (2024) created a nomogram for fresh IVF/ICSI cycles using ovarian response [18]. These studies demonstrate the value of nomograms in guiding treatment and counseling in assisted reproduction.

In this study, we aimed to investigate pregnancy outcomes and develop predictive models, including nomograms, for infertile women with low ovarian reserve (LOR) who received either Chinese herbal medicine (CHM) treatment alone or a combination of IVF and adjuvant CHM therapy. By analyzing patient characteristics and treatment modalities, we sought to provide a reliable tool to estimate the likelihood of achieving a live birth in this specific population.

## 2. Materials and Methods

### 2.1. Data Source and Study Design

A retrospective cohort study was carried out from the outpatient records of Tainan Municipal Hospital (managed by Show Chwan Medical Care Corporation) in Taiwan. The patient data from January 2017 to December 2019 were retrieved. We enrolled patients diagnosed with female infertility by using the ICD-9-CM (the International Classification of Diseases, 9th Revision, Clinical Modification) code 6289. The collected data contained age, duration of infertility, body mass index (BMI), serum AMH levels, smoking, alcohol use, primary or secondary infertility, and causes of infertility. The eligibility criteria for inclusion were: (1) duration of infertility ≥ 1 year; aged 25–45 years; (2) the level of serum AMH < 2.0 ng/mL before treatment; (3) normal tubal patency; (4) partners with normal sperm quality. Exclusion criteria were: (1) loss to follow-up; (2) intrauterine insemination; (3) chromosomal abnormality; (4) uterine malformations; (5) contraceptive pill use or adjuvant supplementation for at least four weeks prior to the study; (6) cancer treatments; and (7) received other medicines, certain micronutrients, and adjuvant supplementation, such as dehydroepiandrosterone (DHEA), coenzyme Q10 (CoQ10) vitamin E, and growth hormone, during the CHM treatment. The following patients were excluded from this study: donor oocytes, sperm, pre-implantation genetic diagnosis (PGD), and pre-implantation genetic diagnosis (PGS).

### 2.2. Interventions

All patient data in our study were retrieved from the outpatient records of the Department of Traditional Chinese Medicine of Tainan Municipal Hospital. Women who received no treatment or acupuncture or nutritional supplements were classified in the control group. Subjects with CHM therapy alone for at least 3 months were assigned to the CHM-only group; other participants who received IVF cycle combined CHM treatment were assigned to the mixed group. Participants in the mixed group received CHM treatment for preparation about 3 months before entry into the first cycle of ovulation induction, and all cases were provided CHM continuously following embryo transfer to support implantation and protect the fetus. Using at least 3 months of CHM treatment was based on the data that primordial follicles enter into a mature oocyte during 90 days. The patient’s condition involved an analysis of the clinical data of symptoms, physical signs, serum AMH levels, phase of the menstrual cycle, and lifestyle factors assessed after full consultation. An individual CHM formula was decided by Chinese medical doctors with at least five years of experience in infertility treatment, based on the theories of TCM.

### 2.3. Evaluation Indexes

The main evaluation standards were clinical pregnancy and live birth rates. Clinical pregnancy was diagnosed as the presence of one or more gestational sac by ultrasonographic visualization (6 to 8 weeks’ gestation). Multiple gestational sacs are counted as one clinical pregnancy. During fresh or frozen embryo transfer IVF treatment, the clinical pregnancy rate was defined as the number of clinical pregnancies divided by the number of embryo transfer cycles. Live birth was determined by the birth of at least one live-born infant of >20 weeks of gestation. During fresh or frozen embryo transfer IVF treatment, the live birth rate was defined as the number of live offspring delivered divided by the number of embryo transfer cycles regardless of whether there are singleton or multiple births, both are considered as only one live birth. The definitions of terms related to infertility and ART are based on the International Committee for Monitoring Assisted Reproductive Technology (ICMART) and World Health Organization glossary of ART terminology [14].

### 2.4. Statistical Analysis

All data analyses were performed using statistical software R 3.6.3 for Windows. The continuous variables (e.g., age, infertility duration, BMI, and AMH levels) of patients were expressed as mean ± standard deviation, and categorical variables (e.g., smokers, alcohol use, infertility type, causes of infertility, and pregnancy outcomes) were expressed as a percentage (%). The comparison in the means of continuous variables between the two three groups was performed using an ANOVA Student’s *t*-test. Categorical variables were evaluated using a chi-square test or Fisher’s exact test. A *p*-value < 0.05 was considered statistically significant. Logistic regression analysis was used to examine the association between pregnancy outcomes and patients’ characteristics. A nomogram according to the regression coefficient based on the relevant variable. The receiver operating characteristic (ROC) curve with area under the curve (AUC) of the live birth rate were performed to evaluate the prediction accuracy of the model. A 95% confidence interval of AUC was also obtained. All statistical tests were two-sided, and a *p*-value less than 0.05 indicates statistical significance.

### 2.5. Ethics

The requirement of patient approval or informed consent was not available, as this study involved a retrospective medical record analysis. This study was approved by the Institutional Review Board (IRB) of the Show Chwan Medical Foundation (Approval Code: IRB No. 1090501; Approval Date: 1 June 2020). The organized IRB is conducted in accordance with Good Clinical Practice, and all applicable laws and regulations.

## 3. Results

### 3.1. Comparison of Baseline Characteristics Among Patients

**Figure 1:**
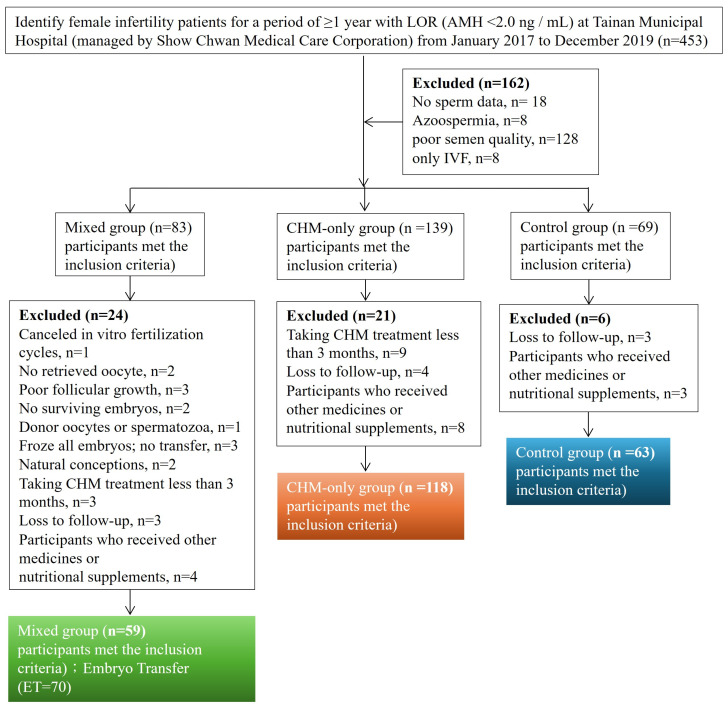
T Flow diagram of the selection of eligible studies.

From January 2017 to December 2019, 240 eligible patients with LOR (AMH < 2.0 ng/mL) and their husbands with normal sperm quality were included in this retrospective study. The study population was divided into three groups, including the control group (*n* = 63), CHM treatment alone (CHM-only group, *n* = 118), and the combination of IVF with CHM treatment (mixed group, *n* = 59) (Figure 1). Baseline demographics are shown in Table 1 and there were no differences among the three groups (*p* > 0.05). The mean ages were 37.63 ± 4.00, 36.69 ± 3.69, and 38.39 ± 4.00 years old, married years were 4.34 ± 3.12, 4.08 ± 2.91, and 4.38 ± 2.73 years, the average BMIs were 22.11 ± 3.22, 22.51 ± 3.71, and 22.54 ± 3.81 kg/m^2^, and the average serum AMH levels were 0.94 ± 0.57, 1.03± 0.55, and 0.93 ± 0.58 ng/mL, proportion of smoking were 2%, 0%, and 0%, proportion of alcohol use were 5%, 3%, and 2% for the control group, CHM-only group, and mixed group, respectively (Table 1). Overalll, age, married year, BMI, AMH level, smoking status, and alcohol use with no significant differences among groups (Table 1).

A majority of participants in all groups had primary infertility (63–68%) and secondary infertility (32–37%), with no significant difference among groups (*p* = 0.879, Table 1). Low ovarian reserve (LOR): This was the universal cause across all participants (100%), thus no variation or statistical comparison was applicable. Polycystic ovary syndrome (PCOS): Present in 1–6% of participants; no significant difference (*p* = 0.341, Table 1). Endometriosis: Found in 17–22% of participants across groups; no significant difference (*p* = 0.813, Table 1). Uterine myoma: Observed in 10–17% of participants; not significantly different (*p* = 0.483, Table 1). Endocrine disorders, immune disorders, and high prolactin levels: Each reported in a small subset of participants, with no significant differences among groups (Table 1).

### 3.2. Univariate Logistic Regression Analysis of Pregnancy Outcomes Among Patients

**Figure 2:**
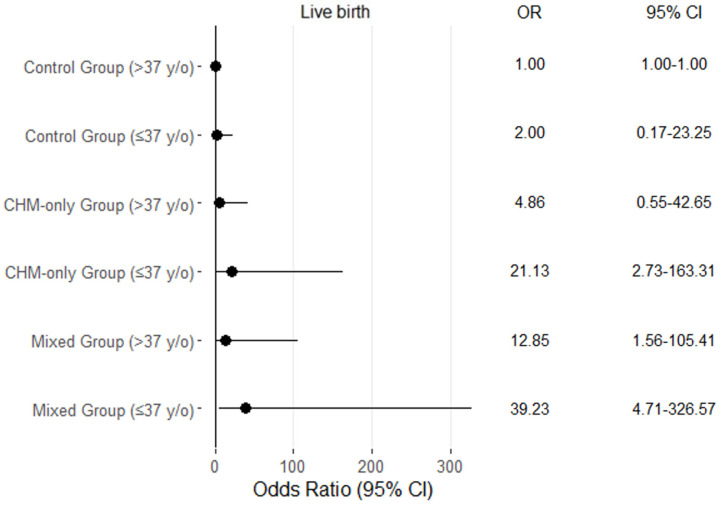
Forest plot showing the odds ratios (ORs) and 95% confidence intervals (CIs) for live birth across different treatment groups and age strata.

Univariate logistic regression analyses were conducted to assess the association of treatment type, age, body mass index (BMI), anti-Müllerian hormone (AMH) levels, and infertility type with clinical pregnancy and live birth outcomes. Compared to the control group, both the CHM-only group and the mixed treatment group showed significantly higher odds of achieving clinical pregnancy and live birth. Specifically, the CHM-only group had significantly increased odds of clinical pregnancy (OR = 4.93, 95% CI: 2.06–11.76, *p* < 0.001, Table 2) and live birth (OR = 9.13, 95% CI: 2.68–31.03, *p* < 0.001, Table 2). The mixed treatment group demonstrated even higher odds for clinical pregnancy (OR = 8.47, 95% CI: 3.39–21.14, *p* < 0.001, Table 2) and live birth (OR = 14.14, 95% CI: 4.04–49.53, *p* < 0.001, Table 2). Younger age (≤37 years) was associated with a significantly greater likelihood of live birth (OR = 2.87, 95% CI: 1.57–5.25, *p* = 0.001, Table 2), although the increase in clinical pregnancy rate did not reach statistical significance (OR = 1.53, 95% CI: 0.90–2.59, *p* = 0.114, Table 2). BMI and AMH levels were not significantly associated with either clinical pregnancy or live birth outcomes. Likewise, secondary infertility was not significantly associated with higher clinical pregnancy (OR = 1.73, 95% CI: 0.90–3.31, *p* = 0.098, Table 2) or live birth rates (OR = 1.19, 95% CI: 0.59–2.40, *p* = 0.621, Table 2). We explored the interaction effect between treatment groups and age by including an interaction term in a logistic regression model (treatment × age group). The results were visualized using a stratified forest plot, as shown in the revised figure. The stratified odds ratios suggest potential effect modification, particularly in the CHM-only and Mixed groups, where younger participants (≤37 y/o) had substantially higher odds of live birth compared to older participants (Table 2 and Figure 2).

### 3.3. Multivariate Logistic Regression Analysis of Pregnancy Outcomes Among Control Group and CHM-Only Group Patients

Multivariate logistic regression was used to evaluate the independent effects of treatment type, age, BMI, AMH levels, and infertility type on the likelihood of achieving clinical pregnancy and live birth. All models were adjusted for these covariates. After adjusting for covariates, the CHM-only group had significantly higher odds of both clinical pregnancy and live birth compared to the control group, suggesting CHM has a strong independent effect. Women ≤37 years had a significantly higher chance of live birth (adjusted OR = 4.65, *p* = 0.002, Table 3). The effect on clinical pregnancy was not statistically significant (*p* = 0.172, Table 3), though a positive trend was observed. BMI was not significantly associated with clinical pregnancy or live birth probability. AMH levels were not independently associated with clinical pregnancy or live birth outcomes in the adjusted model. Secondary infertility was significantly associated with increased clinical pregnancy probability (*p* = 0.030, Table 3). The association with live birth was not statistically significant, although a positive trend was observed.

In summary, after adjusting for age, BMI, AMH, and infertility type, the CHM-only treatment was an independent positive predictor of both clinical pregnancy and live birth. Younger age (≤37 years) and secondary infertility were also positively associated with reproductive outcomes, with significance observed for live birth (age) and clinical pregnancy (infertility type). BMI and AMH levels were not significant predictors in this model.

### 3.4. Multivariate Logistic Regression Analysis of Pregnancy Outcomes Among Control Group and Mixed Group Patients

**Figure 3:**
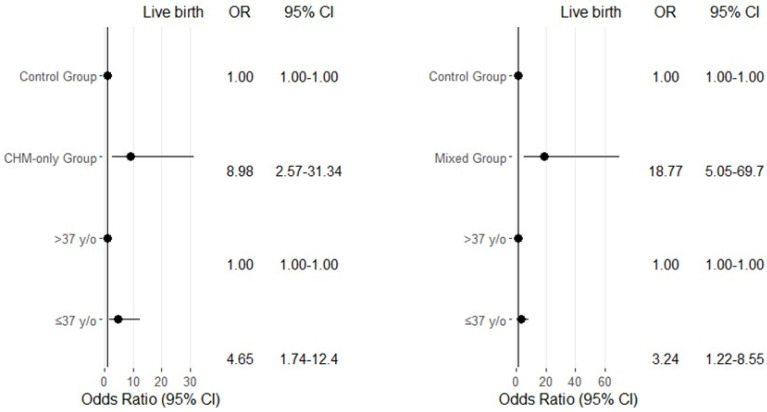
Multivariate logistic regression analysis of the odds ratios for live birth probability among infertile females, focusing on age ≤37 years, CHM-only treatment, and combined (CHM plus conventional) treatment.

Multivariate logistic regression analyses were performed to evaluate the association between treatment type, age, body mass index (BMI), anti-Müllerian hormone (AMH) levels, and infertility type with clinical pregnancy and live birth probabilities (Table 4). Compared to the control group, the mixed treatment group demonstrated a significantly higher likelihood of achieving both clinical pregnancy (adjusted odds ratio [aOR] = 10.61; 95% confidence interval [CI]: 3.96–28.43; *p* < 0.001, Table 4) and live birth (aOR = 18.77; 95% CI: 5.05–69.70; *p* < 0.001, Table 4), indicating a strong positive effect of the mixed treatment approach. Age also showed a significant association with live birth outcomes. Women aged ≤37 years had significantly higher odds of live birth compared to those aged > 37 years (aOR = 3.24; 95% CI: 1.22–8.55; *p* = 0.018, Table 4). However, the association between age and clinical pregnancy did not reach statistical significance (aOR = 1.91; 95% CI: 0.81–4.54; *p* = 0.138, Table 4). In contrast, BMI did not show a significant effect on either clinical pregnancy (aOR = 1.19; 95% CI: 0.50–2.79; *p* = 0.685) or live birth (aOR = 1.34; 95% CI: 0.51–3.52; *p* = 0.542, Table 4). Similarly, AMH levels were not significantly associated with clinical pregnancy (aOR = 0.98; 95% CI: 0.42–2.25; *p* = 0.963, Table 4) or live birth (aOR = 0.55; 95% CI: 0.22–1.41; *p* = 0.216, Table 4). Regarding infertility type, secondary infertility was associated with a higher, though not statistically significant, likelihood of clinical pregnancy compared to primary infertility (aOR = 3.08; 95% CI: 0.98–9.65; *p* = 0.053, Table 4). The difference in live birth rates between secondary and primary infertility groups was also not statistically significant (aOR = 1.76; 95% CI: 0.50–6.17; *p* = 0.375, Table 4). In the left panel, the comparison is between the control group and the Chinese herbal medicine (CHM)-only group. The CHM-only group showed a significantly higher likelihood of live birth compared to the control group, with an OR of 8.98 (95% CI: 2.57–31.34, Figure 3). This indicates that patients who received CHM-only treatment had nearly nine times the odds of achieving a live birth relative to those in the control group. In terms of age, women aged ≤37 years demonstrated significantly greater odds of live birth compared to those older than 37 years (OR = 4.65; 95% CI: 1.74–12.4, Figure 3), suggesting that younger age is a favorable prognostic factor for live birth in this treatment context. In the right panel, the analysis compares the mixed treatment group with the control group. The mixed treatment group exhibited an even stronger association with live birth, with an OR of 18.77 (95% CI: 5.05–69.70, Figure 3), indicating an approximately 19-fold increase in the odds of live birth compared to the control group. As in the right panel, women aged ≤37 years were more likely to achieve a live birth than those over 37 years, with an OR of 3.24 (95% CI: 1.22–8.55, Figure 3), reinforcing the positive impact of younger age on treatment outcomes. In both models, the control group and women aged > 37 years served as reference categories (OR = 1.00, Figure 3). The confidence intervals for the treatment groups were relatively wide, reflecting variability, but all remained above 1.00, indicating statistically significant associations. Overall, the forest plots underscore that both CHM-only and mixed treatment modalities are strongly associated with improved live birth outcomes, and that younger maternal age significantly enhances the likelihood of treatment success.

### 3.5. Prediction of the Live Birth Outcomes Using Nomograms

**Figure 4:**
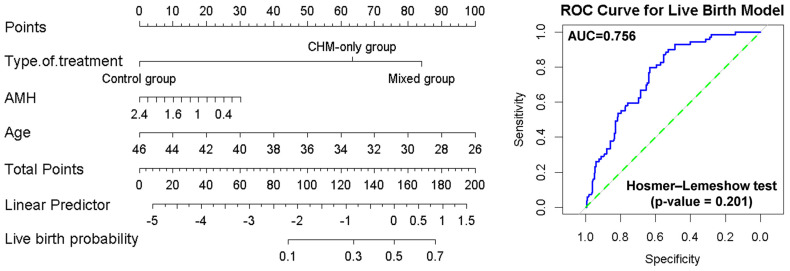
Nomograms for predicting live birth probability in infertile women undergoing CHM-only or mixed treatment. Receiver operating characteristic (ROC) curves of the types of treatment in live birth. The ROC curve evaluates the discriminative performance of the logistic regression model used to construct the nomogram. The blue curve represents the model, and the green diagonal line corresponds to a non-informative model with an area under the curve (AUC) of 0.5 and Hosmer–Lemeshow test (*p*-value = 0.201).

The nomogram integrates three significant predictors—type of treatment, anti-Müllerian hormone (AMH) level, and age—to estimate the individual probability of achieving a live birth (Figure 4). Each predictor corresponds to a specific number of points on the topmost “Points” scale. For the type of treatment, patients receiving mixed treatment receive the highest point contribution, followed by those in the CHM-only group, with the control group receiving zero points, reflecting its reference status in the model (Figure 4). AMH levels are inversely associated with points: lower AMH (closer to 0 ng/mL) corresponds to higher points and thus lower predicted live birth probability (Figure 4). Younger age also contributes more points, indicating that younger patients have higher predicted probabilities of live birth (Figure 4). By summing the individual point values for all predictors, a total point score is obtained. This total is then mapped to a linear predictor scale, which is further translated into the predicted live birth probability at the bottom of the nomogram, ranging approximately from 0.1 to 0.7 (Figure 4). The AUC of the model is 0.756 and the Hosmer–Lemeshow test has a *p*-value = 0.201, indicating good discriminatory power. This means the model has a 75.6% chance of correctly distinguishing between individuals who will and will not achieve a live birth. The upward bend of the ROC curve toward the top-left corner reflects favorable sensitivity and specificity trade-offs.

## 4. Discussion

This retrospective study evaluated the impact of Chinese herbal medicine (CHM), both as a standalone treatment and in combination with in vitro fertilization (IVF), on clinical pregnancy and live birth outcomes in women with low ovarian reserve (LOR). Our findings demonstrate that both CHM-only and mixed treatment modalities significantly improve reproductive outcomes compared to no treatment. These results have important clinical implications, especially for women with diminished ovarian reserve who have limited treatment options. Our univariate and multivariate logistic regression analyses consistently showed that CHM-only treatment significantly increased the likelihood of both clinical pregnancy and live birth. Furthermore, when CHM was combined with IVF, the positive effect on outcomes was even more pronounced. Compared with the control group, the CHM-only group had an 8.98-fold increase in live birth odds, while the mixed group showed an 18.77-fold increase, highlighting the synergistic potential of combining traditional and assisted reproductive techniques.

Age also emerged as a significant predictor of live birth, with women aged ≤37 years consistently showing higher success rates. This aligns with the well-established understanding that younger maternal age is associated with better ovarian function and endometrial receptivity, even among women with LOR. Interestingly, traditional predictors such as body mass index (BMI) and serum anti-Müllerian hormone (AMH) levels were not significantly associated with clinical pregnancy or live birth outcomes in adjusted models. This may reflect the relatively narrow range of AMH values in the study population due to the inclusion criterion (AMH < 2.0 ng/mL), which may limit its predictive power in this specific context. Similarly, BMI was within a generally normal range for most participants, possibly limiting its discriminatory capacity. A woman’s age plays a crucial role in determining pregnancy outcomes. Several studies have shown that pregnancy rates decline progressively as female age increases [19,20]. In our study, the AMH levels in the nomogram represent pretreatment values collected before CHM or IVF. The inverse relationship indicates that lower baseline AMH is associated with higher predicted live birth probabilities, possibly reflecting a role of CHM in improving AMH levels or ovarian function.

The type of infertility (primary vs. secondary) showed some association with outcomes, particularly with clinical pregnancy. Secondary infertility was positively associated with pregnancy outcomes in some models, though not consistently across all analyses. This may suggest that women who have previously conceived may retain a higher chance of subsequent pregnancy despite LOR.

In recent years, the rate of ovarian function degeneration has gradually increased, which is related to the continuous socioeconomic development and changes in people’s lifestyle, work pace, and diet structure. Age is one of the most important factors affecting ovarian function in women, and ovarian reserve function decreases gradually after 30 years of age, especially after 35 years of age [21,22]. In addition, the high levels of stress associated with modern work and life often lead to metabolic disorders and poor blood circulation in women, which may be major contributing factors to infertility. Currently, the integration of Chinese and Western medicine is becoming an increasingly popular approach, particularly in the treatment of chronic conditions and gynecological disorders such as infertility.

Although several predictive models, such as nomograms, have been developed to estimate pregnancy rates in infertile patients [23,24,25], none of the existing predictive models are suitable for assessing pregnancy outcomes in patients with low ovarian reserve (LOR) who receive either Chinese herbal medicine (CHM) alone or CHM as an adjuvant to IVF treatment. In this study, we developed a nomogram based on data from 240 infertile patients with LOR to predict individual clinical pregnancy rates across three treatment approaches: no treatment, CHM alone, and IVF combined with CHM. Our analysis identified three independent factors—female age, type of treatment, and serum AMH levels—that were significantly associated with live birth rates (Figure 3).

The mechanisms by which Chinese herbal medicine (CHM) improves fertility in patients with low ovarian reserve (LOR) involve several biological pathways that support ovarian function and enhance the likelihood of pregnancy. These include the reduction of elevated follicle-stimulating hormone (FSH) and luteinizing hormone (LH) levels, which are often associated with impaired ovarian activity [25,26]. CHM has also been shown to increase antral follicle count (AFC), an important marker of ovarian reserve [27,28], and elevate serum estradiol (E2) levels, which are essential for endometrial development and ovulation [26,27]. Additionally, CHM helps alleviate inflammation in ovarian tissues [28], creating a more favorable environment for follicular development. It also plays a protective role by preventing apoptosis of ovarian granulosa cells [27], which are critical for oocyte maturation and hormone production. Through these combined effects, CHM contributes to improving both ovarian reserve and pregnancy outcomes in women with LOR. Some potential mechanisms of CHM in IVF for improving the success rate have been proposed [29,30,31]: (1) increasing ovarian perfusion; (2) improving the quality of oocytes or embryos; (3) increasing endometrial blood flow in the uterus; (4) alleviating anxiety and stress of infertile patients; and (5) promoting early embryonic development. After CHM pretreatment on the patients with LOR undergoing IVF cycles in the study, clinical pregnancy rate was 52% and live birth rate was 42% (Table 2). Hence, this study demonstrated the beneficial effects of CHM for patients with LOR undergoing IVF cycles.

### Limitations

The retrospective design may introduce selection bias and unmeasured confounding. Treatment allocation lacked randomization and was likely influenced by patient preference or financial factors, contributing to potential bias. Although the overall sample size supported statistical analysis, small subgroup sizes (e.g., PCOS or uterine myoma) limited comparative analyses. The individualized nature of CHM prescriptions based on TCM diagnoses may affect reproducibility. Baseline demographics were comparable, but key treatment-related variables, such as stimulation protocols and embryo quality were not consistently documented and thus excluded, potentially confounding outcomes, particularly in the IVF group. Patients who selected CHM-only treatment may differ from those undergoing IVF in unmeasured aspects such as disease severity or treatment preference, introducing additional bias. We adjusted for key clinical variables (age, BMI, AMH, infertility type), but residual confounding may persist. IVF-specific factors (e.g., oocyte yield, fertilization rates, embryo quality, endometrial thickness, luteal support) are important for outcome prediction and should be included in future models. However, these data were not consistently available in the current study due to the retrospective nature and incomplete documentation in the mixed group.

## 5. Conclusions

The significant reproductive benefits observed with CHM treatment, particularly when combined with IVF, suggest that CHM may offer a valuable adjunctive strategy for patients with LOR. These findings support further investigation into the mechanisms by which CHM may enhance ovarian responsiveness or endometrial receptivity. Moreover, future prospective, randomized controlled trials are warranted to validate the observed effects and minimize potential biases inherent in retrospective analyses. The predictive nomogram developed in this study may serve as a preliminary tool for clinicians to estimate live birth probability in LOR patients and facilitate shared decision making. Future work could incorporate additional biomarkers and psychosocial factors to refine prediction accuracy.

## Figures and Tables

**Table 1:** Baseline characteristics between the mixed group, CHM-only group, and control group.

Variable	Control Group	CHM-Only Group	Mixed Group	*p*-Value
	(*n* = 63)	(*n* = 118)	(*n* = 59) ET = 70	
Age, years (SD)	37.63 (4.00)	36.69 (3.69)	38.39 (4.00)	0.250
Married year, year (SD)	4.34 (3.12)	4.08 (2.91)	4.38 (2.73)	0.764
BMI, mean (SD)	22.11 (3.22)	22.51 (3.71)	22.54 (3.81)	0.734
AMH levels (ng/mL)	0.94 (0.57)	1.03 (0.55)	0.93 (0.58)	0.410
Smokers				
No	62 (98%)	118 (100%)	59 (100%)	0.244
Yes	1 (2%)	0 (0%)	0 (0%)	
Alcohol use				
No	60 (95%)	115 (97%)	58 (98%)	0.569
Yes	3 (5%)	3 (3%)	1 (2%)	
Infertility type				
Primary	40 (63%)	78 (66%)	40 (68%)	0.879
Secondary	23 (37%)	40 (34%)	19 (32%)	
Causes of infertility				
LOR	63 (100%)	118 (100%)	59 (100%)	ND
PCOS	1 (1%)	7 (5%)	4 (6%)	0.341
Endometriosis	11 (17%)	24 (20%)	13 (22%)	0.813
Uterine myoma	9 (14%)	20 (17%)	6 (10%)	0.483
Endocrine disorders	4 (6%)	11 (9%)	7 (11%)	0.571
Immune disorder	2 (3%)	4 (3%)	3 (5%)	0.822
High prolactin levels	6 (10%)	11 (9%)	7 (11%)	0.859

ET, embryo transfer. BMI, body mass index; AMH, anti-Müllerian hormone; LOR, low ovarian reserve; PCOS, polycystic ovarian syndrome. *p*-values are based on t test for continuous variables and chi-square tests or Fisher exact test for categorical variables. ND, not detectable.

**Table 2:** Univariate logistic regression analyses of type of treatment, age, BMI, AMH, and infertility type for clinical pregnancy probability and live birth probability.

		Clinical Pregnancy Rate, n (%)				Live Birth Rate, n (%)			
Type of Treatment		No	Yes	OR, 95% CI	*p* Value	No	Yes	OR, 95% CI	*p* Value
Control group	63	56 (88)	7 (12)	Reference		60 (95)	3 (5)	Reference	
CHM-only group	118	73 (62)	45 (38)	4.93 (2.06–11.76)	<0.001	81 (68)	37 (32)	9.13 (2.68–31.03)	<0.001
Mixed group (ET)	70	34 (48)	36 (52)	8.47 (3.39–21.14)	<0.001	41 (58)	29 (42)	14.14 (4.04–49.53)	<0.001
Age, years									
>37 (medium)	114	80 (70)	34 (30)	Reference		95 (83)	19 (17)	Reference	
≤37 (medium)	137	83 (60)	54 (40)	1.53 (0.90–2.59)	0.114	87 (63)	50 (37)	2.87 (1.57–5.25)	0.001
BMI, kg/m^2^									
≤21.78 (medium)	126	84 (66)	42 (34)	Reference		91 (72)	35 (28)	Reference	
>21.78 (medium)	125	79 (63)	46 (37)	1.16 (0.69–1.65)	0.565	91 (72)	34 (28)	0.97 (0.55–1.69)	0.918
AMH levels (ng/mL)									
≤1.01 (medium)	126	80 (63)	46 (37)	Reference		86 (68)	40 (32)	Reference	
>1.01 (medium)	125	83 (66)	42 (34)	0.88 (0.52–1.47)	0.629	96 (76)	29 (24)	0.64 (0.37–1.13)	0.131
Infertility type									
Primary	205	138 (67)	67 (23)	Reference		150 (73)	55 (27)	Reference	
Secondary	46	25 (54)	21 (46)	1.73 (0.90–3.31)	0.098	32 (69)	14 (31)	1.19 (0.59–2.40)	0.621
Type of Treatment* Age									
Control group (>37)	31	27 (87)	4 (13)	Reference		30 (96)	1 (4)	Reference	
Control group (≤37)	32	29 (90)	3 (10)	0.69 (0.14–3.41)	0.657	30 (93)	2 (7)	2.00 (0.17–23.25)	0.580
CHM-only group (>37)	43	30 (69)	13 (31)	2.92 (0.85–10.06)	0.089	37 (86)	6 (14)	4.86 (0.55–42.65)	0.153
CHM-only group (≤37)	75	43 (57)	32 (43)	5.02 (1.59–15.79)	0.006	44 (58)	31 (42)	21.13 (2.73–163.31)	0.003
Mixed group (>37)	40	23 (57)	17 (43)	4.98 (1.46–16.94)	0.010	28 (70)	12 (30)	12.85 (1.56–105.41)	0.017
Mixed group (≤37)	30	11 (36)	19 (64)	11.65 (3.22–42.19)	<0.001	13 (43)	17 (57)	39.23 (4.71–326.57)	0.001

**Table 3:** Multivariate logistic regression analyses of type of treatment (control group and CHM-only group), age, BMI, AMH, and infertility type for clinical pregnancy probability and live birth probability.

		Clinical Pregnancy Rate, n (%)				Live Birth Rate, n (%)			
Type of Treatment		No	Yes	OR, 95% CI	^a^ *p* Value	No	Yes	OR, 95% CI	^a^ *p* Value
Control group	63	56 (88)	7 (12)	Reference		60 (95)	3 (5)	Reference	
CHM-only group	118	73 (62)	45 (38)	5.04 (2.06–12.31)	<0.001	81 (68)	37 (32)	8.98 (2.57–31.34)	0.001
Age, years									
>37 (medium)	74	57 (77)	17 (23)	Reference		67 (90)	7 (10)	Reference	
≤37 (medium)	107	72 (67)	35 (33)	1.69 (0.79–3.62)	0.172	74 (69)	33 (31)	4.65 (1.74–12.40)	0.002
BMI, kg/m^2^									
≤21.78 (medium)	93	66 (70)	27 (30)	Reference		71 (76)	22 (24)	Reference	
>21.78 (medium)	88	63 (71)	25 (29)	0.90 (0.44–1.83)	0.787	70 (79)	18 (21)	0.89 (0.40–1.98)	0.784
AMH levels (ng/mL)									
≤1.01 (medium)	93	64 (68)	29 (32)	Reference		69 (74)	24 (26)	Reference	
>1.01 (medium)	88	65 (73)	23 (25)	0.80 (0.39–1.69)	0.532	72 (81)	16 (19)	0.62 (0.28–1.37)	0.238
Infertility type									
Primary	145	108 (74)	37 (26)	Reference		115 (79)	30 (21)	Reference	
Secondary	36	21 (58)	15 (42)	2.59 (1.09–6.15)	0.030	26 (72)	10 (28)	2.27 (0.82–6.28)	0.112

^a^: Adjusted for type of treatment, age, BMI, AMH1, and infertility type.

**Table 4:** Multivariate logistic regression analyses of type of treatment (control group and mixed group), age, BMI, AMH, and infertility type for clinical pregnancy probability and live birth probability.

		Clinical Pregnancy Rate, n (%)				Live Birth Rate, n (%)			
Type of Treatment		No	Yes	OR, 95% CI	^a^ *p* Value	No	Yes	OR, 95% CI	^a^ *p* Value
Control group	63	56 (88)	7 (12)	Reference		60 (95)	3 (5)	Reference	
Mixed group (ET)	70	34 (48)	36 (52)	10.61 (3.96–28.43)	<0.001	41 (58)	29 (42)	18.77 (5.05–69.70)	<0.001
Age, years									
>37 (medium)	71	50 (70)	21 (30)	Reference		58 (81)	13 (19)	Reference	
≤37 (medium)	62	40 (64)	22 (36)	1.91 (0.81–4.54)	0.138	43 (69)	19 (31)	3.24 (1.22–8.55)	0.018
BMI, kg/m^2^									
≤21.78 (medium)	68	48 (70)	20 (30)	Reference		53 (77)	15 (23)	Reference	
>21.78 (medium)	65	42 (64)	23 (36)	1.19 (0.50–2.79)	0.685	48 (73)	17 (27)	1.34 (0.51–3.52)	0.542
AMH levels (ng/mL)									
≤1.01 (medium)	68	47 (69)	21 (31)	Reference		50 (73)	18 (27)	Reference	
>1.01 (medium)	65	43 (66)	22 (34)	0.98 (0.42–2.25)	0.963	51 (78)	14 (22)	0.55 (0.22–1.41)	0.216
Infertility type									
Primary	110	77 (70)	33 (30)	Reference		84 (76)	26 (24)	Reference	
Secondary	23	13 (56)	10 (44)	3.08 (0.98–9.65)	0.053	17 (73)	6 (27)	1.76 (0.50–6.17)	0.375

^a^: Adjusted for type of treatment, age, BMI, AMH1, and infertility type.

## Data Availability

The data presented in this study are available on request from the corresponding author.

## Abbreviations

The following abbreviations are used in this manuscript:

DOR	Diminished ovarian reserve
IVF	In vitro fertilization
AMH	Anti-mullerian hormone
CHM	Chinese herbal medicine
ET	Embryo transfer
